# Dementia Care Coordination Program Implementation Evaluation

**DOI:** 10.1002/alz70858_110765

**Published:** 2025-12-26

**Authors:** Nancy A Hodgson

**Affiliations:** ^1^ University of Pennsylvania, Philadelphia, PA, USA

## Abstract

Dementia Care Coordination (DCC) represents a novel telephone‐based care coordination service that bridges the gap between clinical care and community support by training healthcare providers to systematically identify dementia caregivers and refer them to trained Alzheimer's Association care consultants who provide personalized education, resources, and ongoing support. This innovative model addresses critical care coordination gaps in dementia care delivery. Caregivers demonstrated maintained or improved self‐efficacy scores following DCC participation. Despite experiencing common barriers including caregiver burden and time constraints, participants showed exceptionally high engagement: 70% action plan uptake, 80% resource utilization, and 90% action plan comprehension. Health system employees reported high program satisfaction, though clinician engagement barriers were identified. The DCC model demonstrates promising effectiveness in coordinating dementia care across multiple geographic regions and diverse health system contexts. This multi‐state implementation demonstrates the feasibility and promise of innovative telephone‐based dementia care coordination. The rigorous implementation science evaluation identified key success factors and improvement opportunities, providing evidence‐based guidance for program refinement, sustainability planning, and strategic expansion to additional healthcare systems and regions.

